# Excel Methods to Design and Validate in Microelectronics (Complementary Metal–Oxide–Semiconductor, CMOS) for Biomedical Instrumentation Application

**DOI:** 10.3390/s21227486

**Published:** 2021-11-11

**Authors:** Graciano Dieck-Assad, José Manuel Rodríguez-Delgado, Omar Israel González Peña

**Affiliations:** 1 Tecnológico de Monterrey, School of Engineering and Science, Av. Eugenio Garza Sada Sur No. 2501, col. Tecnológico, Monterrey 64849, Mexico; graciano.dieck.assad@tec.mx (G.D.-A.); jmrd@tec.mx (J.M.R.-D.); 2 Tecnológico de Monterrey, Institute for the Future of Education, Av. Eugenio Garza Sada Sur No. 2501, col. Tecnológico, Monterrey 64849, Mexico

**Keywords:** freeware, open science, analog microelectronics design, long channel transistors, short channel transistors, integrated circuit design, CMOS design, VLSI, higher education, educational innovation, integrated circuit layout, complex thinking

## Abstract

CMOS microelectronics design has evolved tremendously during the last two decades. The evolution of CMOS devices to short channel designs where the feature size is below 1000 nm brings a great deal of uncertainty in the way the microelectronics design cycle is completed. After the conceptual idea, developing a thinking model to understand the operation of the device requires a good “ballpark” evaluation of transistor sizes, decision making, and assumptions to fulfill the specifications. This design process has iterations to meet specifications that exceed in number of the available degrees of freedom to maneuver the design. Once the thinking model is developed, the simulation validation follows to test if the design has a good possibility of delivering a successful prototype. If the simulation provides a good match between specifications and results, then the layout is developed. This paper shows a useful open science strategy, using the Excel software, to develop CMOS microelectronics hand calculations to verify a design, before performing the computer simulation and layout of CMOS analog integrated circuits. The full methodology is described to develop designs of passive components, as well as CMOS amplifiers. The methods are used in teaching CMOS microelectronics to students of electronic engineering with industrial partner participation. This paper describes an exhaustive example of a low-voltage operational transconductance amplifier (OTA) design which is used to design an instrumentation amplifier. Finally, a test is performed using this instrumentation amplifier to implement a front-end signal conditioning device for CMOS-MEMS biomedical applications.

## 1. Introduction

**Figure 1:**
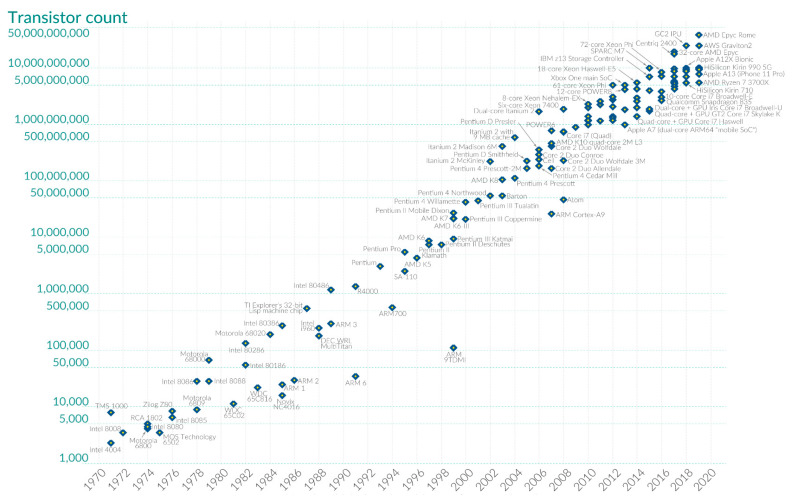
Behavior of the technological development for the small-scale design of transistors on microchips doubling every two years (Moore’s law) [1].

Currently, we have high-capacity technology of analog and digital electronic devices due to the micro-components that are increasingly becoming smaller in scale. In fact, in 1965, Gordon E. Moore predicted (Moore’s law) that every 2 years the number of transistors in a microprocessor would double. This law worked for the first 10 years [1]; then it became a joke between engineers who said that “now people predict that the end of Moore’s law doubles every 2 years.” Since then, transistor integration has been observed as illustrated in Figure 1, where Moore’s law was in force for a long time. However, the limits towards a scale in nanotechnology have shown that although Moore’s law no longer applies, significant efforts are still being made to continue reducing the size of the micro and nano components. For example, recently design and process in semiconductors have been done with the development of the world’s first chip with 2 nanometers (nm) nanosheet technology [2].

Therefore, with this context in which the microchip technology is reducing in size over time, college students with different science and engineering majors need to understand the “know-how” of designing circuits that integrate microchips. Unfortunately, often this knowledge is taught with specialized complex (and expensive) software, which is sometimes not readily available in many universities around the world.

Analog and digital integrated circuit design has emphasized a good understanding of analog and digital electronic circuits to model, simplify, analyze, and simulate microelectronic devices before developing layouts and sending devices to IC fabrication foundry consortiums. Design engineers find SPICE simulations useful to validate “thinking models” [3,4] to verify cause–effect relationships and to ensure that assumptions made are appropriate in the design process. Microelectronics courses and specialized workshops (and webinars) teach conceptual design where the following concepts are emphasized.

- The importance of the integrated circuit (IC) design in microelectronics engineering is embedded into electronic engineering innovation and design flow.
- Analog CMOS microelectronics focus on the electrical and physical design processes.

IC design is important in device development for microelectronics engineering innovation and is embedded into the design flow which may have continuous iterations to optimize designs by decision refinements. In the process of CMOS microelectronics conceptual design [5,6,7], the electrical device specification requires active and passive models for creating, verifying, and determining the robustness of the design. This process involves the selection of a conceptual circuit, the analysis of the selected circuit, the possibility of a modification to the circuit, and the verification of the circuit solution. The physical electronics design process [8,9,10] consists of representing the electrical device in a 2D layout consisting of many different geometrical rectangles at various levels (layers). This layout is then used to implement a 3D integrated circuit during the fabrication process. The device conceptual model follows a process to obtain a layout. This process includes [3,4]:

1. The W/L (width/length of transistors) values and schematic (usually from SPICE simulations).
2. A computer-aided tool (CAD) system is used to enter geometries.
3. The engineer must obey a set of rules called design rules. These rules establish the fabrication limitations and ensure that the device is robust and reliable.
4. Once the layout is complete, a process called layout versus schematic (LVS) is applied to determine if the physical layout represents the electrical schematic.
5. Parasitic elements are extracted, once the physical dimensions of the designs are known. They include capacitances from conductor to ground or between conductors and bulk resistances.
6. The parasitic elements are entered into the simulation database and the design is re-stimulated to ensure they will not cause a device failure.

**Figure 2:**
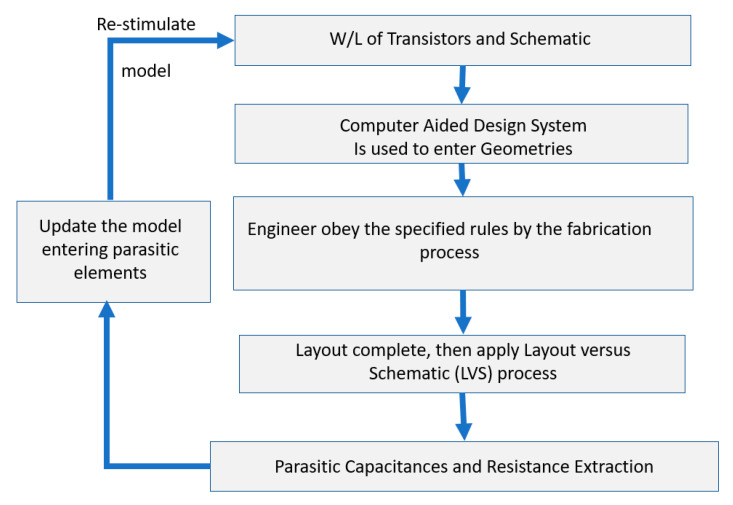
Block diagram showing the design procedure to reach the Layout conceptual model.

This process is depicted in the flow diagram shown below in Figure 2.

Once the first approach to design is terminated, the process continues with design testing which consists of coordinating, planning, and implementing the measurement of the integrated circuit performance. The objective is to compare the experimental performance with the specifications and/or simulation results. Several tests are available, e.g., functional: verification of the nominal specifications; parametric: verification of the characteristics to within a specified tolerance, verification of the static (AC and DC) characteristics of the circuit or system, and verification of the dynamic (transient) characteristics of a circuit or system. Additional testing could include device testing performed at the wafer level or package level and detailed testing that removes the influence of measurement system in the device performance. 

The conceptual design of analog integrated circuits in new devices is now shaped by rules such as: consumers generate a need for new integrated circuits, design engineers have an open possibility of participating in designs, time to develop a product is reduced, profit in products and prototypes are not readily necessary, and the new concept of “crowd designing” [3] plays an important role in device development. This article discusses Excel methods to perform “paper and pencil” calculations (“thinking model”) in the conceptual design of CMOS analog integrated circuits which are validated using ELECTRIC-LTSpice to provide the initial characteristics of the device. Several methodologies have been developed for CMOS device design and testing but most of them are very specific to the final prototype or experimental application [11,12,13,14,15,16,17]. The Excel methods analyzed in this study focus on providing didactic instruction in microelectronics for undergraduate and graduate students. Therefore, it seeks to focus learning by referring to world trends in conducting open science, providing the social appropriation of knowledge. The forefront is didactic management whose central axis is the catalyst of open innovation processes that have proven to be very successful disruptive models in open laboratories, universities, research centers, industry, and government for the development of emerging economies and public policies [18,19,20,21,22,23,24,25,26].

As a result, this study describes a method that uses an Excel spreadsheet to start the conceptual design from the point of view of the “thinking model” or the first cut evaluation of the design with “paper and pencil”. In addition, several contributions stand out:

The Excel methods discussed here focus on microelectronics education for undergraduate and graduate students. The article describes a simple method using an Excel spreadsheet to initiate conceptual design from the standpoint of “thinking model” or “paper and pencil” first cut evaluation of the design. Furthermore, several contributions are emphasized:

(a) This work describes both the conceptual design evaluations using the traditional equations and prepares the way for the layout implementation by setting up the schematic of the design.
(b) In addition, this study describes that once the schematic simulation agrees with the scheduled specifications and the “thinking model” provides a possible design solution, the layout is developed and a comparison between the output results with the thinking model specs validates the third phase of the process.
(c) Moreover, the methodology applied in this study can be developed for more complicated CMOS analog integrated circuits (IC) conceptual designs, specifically for teaching purposes.
(d) Complex conceptual designs can also be addressed using simple and readily available software (freeware) to teach with an open science view.
(e) Furthermore, this article illustrates an exhaustive example of a low voltage operational transconductance amplifier (OTA) design for portable biomedical applications. The test is performed using this instrumentation amplifier to implement a front-end signal conditioning device for CMOS-MEMS biomedical applications.

This manuscript is organized as follows. Section 2 briefly describes device modeling to represent transistors using first principle equations that predict their behavior in different regimes and operating conditions. Section 3 provides the basic MOSFET modeling equations, starting with the threshold voltage calculations, the transconductance equations for the ohmic (sub-saturation), active (saturation), and subthreshold conditions, both for long channel and short channel devices. Section 3 also gives the typical MOSFET parameters for 0.5 mm CMOS technology, the small-signal model parameter equations, and useful resistance and capacitance calculations using the foundry process parameters for the technology. Section 4 describes the Excel methods that students use to develop their first cut approximations in conceptual designs of CMOS devices and before testing schematic simulations and layouts. This section explains three Excel methodologies: single straight, tabular straight, and two-dimensional processing methods to perform the evaluation of the device’s conceptual design. This section also discusses resistance, capacitance, and differential amplifier conceptual designs as specific examples to apply the Excel methods. Section 5 discusses the Excel methods applied for complete amplifier design. Here the cascode amplifier and the OTA (Operational Transconductance Amplifier) are used as examples of how students use the basic two-dimensional Excel methodology to solve CMOS microelectronic conceptual design devices. Section 6 illustrates a complete case study, developed by students and wrapped up by their instructor, of a low-power high-gain operational amplifier for biomedical applications. In this section, comparison of the design is performed with respect to a particular device appearing in the technical literature and an application of a CMOS-MEMS signal conditioning is developed considering the requirement of conditioning a differential mode temperature sensor´s output to obtain a readily available low voltage representation of the temperature of a micro hotplate multisensor platform. Finally, Section 7 wraps up the paper with illustrative conclusions about how these Excel methods have provided extraordinary insights to undergraduate students in their effort to consolidate a good understanding of microelectronics conceptual integrated circuit (IC) design.

## 2. Materials and Methods—Device Modeling

The process of device modeling consists of representing the electrical properties of devices using mathematical equations, circuits, graphs, correlations, and energy conservation laws. Models allow predictions and validation of circuit performance with uncertainties coming from no-idealities and non-linear behaviors in electronic components. Typical equations and conservation laws are Ohm´s law, large- and small-signal models of MOSFET transistors, VTC curves, and I-V curves of diodes. The final goals are to simplify the cause–effect relationships allowing the engineer to understand and consider decisions that increase performance in the circuit. 

**Figure 3:**
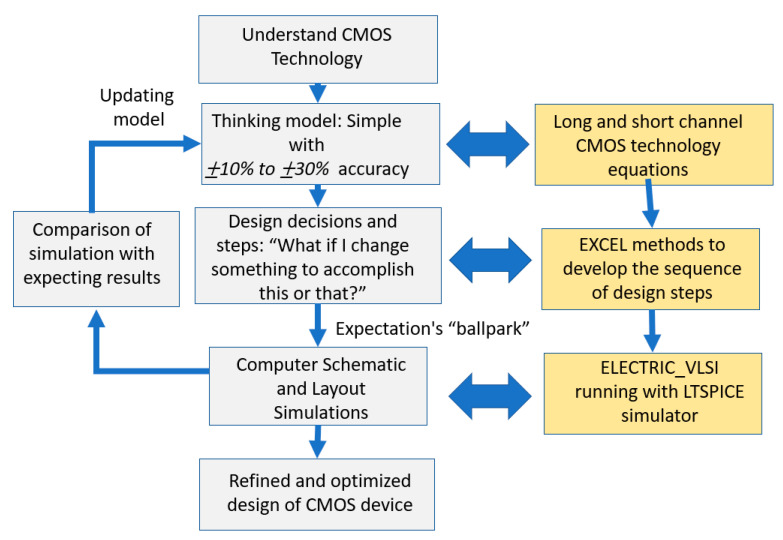
The CMOS microelectronics design process and the insight provided by the Excel methods in the quest to teach microelectronics design with simple tools.

Analog integrated circuits in microelectronic conceptual design are developed using a non-hierarchical structure where the use of repeated blocks is only possible in few devices, and therefore the design process is complex and challenging. To handle this, design engineers use hierarchy whenever possible, use good organization techniques, efficiently document the design, provide reasonable and reliable assumptions and simplifications, and eventually validate the conceptual designs using simulation experiments. Assumptions and simplifications are used to emphasize the essential characteristics by neglecting the non-dominant effects in the design. The challenge of teaching microelectronics is to develop an insight into the design process without requiring specialized professional software which is not readily available in many universities around the world. Figure 3 shows the microelectronics design process, on the left side, with the insight given on the right side, using Excel methods and other simulation techniques which are readily available to universities. 

Analog integrated circuit design and device evaluation have reached a level of maturity in established applications such as digital to analog and analog to digital conversion systems, front end signal conditioning devices, instrumentation channel devices, bandgap reference sources, DC to DC power conversion drives, and other important microelectronic circuits [3]. Finally, analog circuit conceptual designs have significant applications in devices where speed and power have an overwhelming advantage over digital devices. This paper reviews the long and short channel models for CMOS devices and further application of long channel equations using Excel methods for first cut approximations before computer simulations and layout development. Those approximation models can be found in many CMOS microelectronics specialized textbooks [3,4,7,27,28,29] and they are summarized here for completeness in this discussion. 

## 3. Results—Transistor Models for Analog Conceptual Design

### 3.1. Threshold Voltage Calculation

The evaluation of threshold voltage, *V_T_*, is fundamental in the development of CMOS microtechnology because gives the necessary condition for allowing operation of the transistors in the right zone.

(1) $$V_{T}=\left[V_{T0}\right]+\gamma \left(\sqrt{\left|2\varphi_{F}\right|+V_{SB}}-\sqrt{\left|2\varphi_{F}\right|}\right)$$

where *γ* is the body factor, $2\varphi_{F}$ is the Fermi potential, *V_T_*_0_ is the zero bias threshold voltage, and *V_SB_* is the potential difference between source and bulk of the device. 

### 3.2. Current Equations for Long Channel Devices

The drain-source current equations for the MOSFET long channel device are given below for *V_GS_ ≥ V_T_* and *k = μC_ox_(W/L)*:

Saturation zone: *V_DS_ ≥ V_GS_* − *V_T_*

(2) $$i_{D}=\frac{k}{2}{\left[V_{GS}-V_{T}\right]}^{2}{\left(1+\lambda V_{DS}\right)}_{\;}$$

Ohmic zone: *V_DS_ ≤ V_GS_* − *V_T_*

(3) $$i_{D}=\frac{k}{2}\left[2\left(V_{GS}-V_{T}\right)-V_{DS}\right]V_{DS}$$

For *V_GS_* ≤ *V_T_* the transistor plays in the subthreshold zone with the following exponential behavior, like the bipolar junction transistor:

(4) $$i_{D}=I_{t}\frac{W}{L}e^{\frac{\left(V_{GS}-V_{T}\right)}{nV_{t}}}$$

where the nomenclature and parameters of the model are as follows: *i_D_* is the drain to source current flowing in the MOSFET, *μ* is the charge carrier mobility, *C_ox_* is the SiO_2_ oxide capacitance, *(W/L)* is the width/length transistor ratio, *V_GS_* is the gate to source potential differences, *V_DS_* is the drain to source potential differences, *λ* is the transistor´s channel modulation parameter, *I_t_* is the subthreshold saturation current (<1 μA), *V_t_* is the thermal voltage (~26 mV at 25 °C), and n is a subthreshold constant (~1 to 2). 

### 3.3. Velocity Saturation and Effective Mobility in Current Equations of Short Channel Devices

To consider velocity saturation and effective mobility in short channel MOSFETs adjustments are made in the model equations presented above. This is particularly important in the saturated and ohmic regions of operation. The drain-source current equations for the MOSFET short channel device are given below for *V_GS_ ≥ V_T_*, having saturation velocity *v_sat_ = μ_e_ E_c_/2*, where *μ_e_* is now the effective mobility of charge carriers and *E_c_* is the critical field for which the carrier saturation occurs.

Saturation zone:

(5) $$V_{DS}\left(sat\right)=\frac{\left(V_{GS}-V_{T}\right)E_{c}L}{\left(V_{GS}-V_{T}\right)+E_{c}L}$$

(6) $$i_{D}=\frac{W\mu_{e}C_{ox}E_{c}}{2}\left[\frac{{\left(V_{GS}-V_{T}\right)}^{2}}{\left(V_{GS}-V_{T}\right)+E_{c}L}\right]$$

Ohmic zone:

(7) $$V_{DS}\left(ohmic-limit\right)=V_{DS}\left(sat\right)=\frac{\left(V_{GS}-V_{T}\right)E_{c}L}{\left(V_{GS}-V_{T}\right)+E_{c}L}$$

(8) $$i_{D}=\frac{W\mu_{e}C_{ox}E_{c}}{\left(E_{c}L+V_{DS}\right)}\left[\left(V_{GS}-V_{T}\right)V_{DS}-\frac{V_{DS}}{2}\right]V_{DS}$$

If we summarize the SPICE-level I models of the MOSFET transistors to be used in conceptual CMOS designs, Table 1 describes the equations considering the ohmic and saturation regions in the so-called strong inversion regime. 

Moreover, for “paper and pencil” calculations (“Thinking Modeling”) the parameters used for 0.5 μm CMOS technology are described in Table 2.

### 3.4. Small-Signal Model of Devices

The useful small-signal equations for MOSFET transistors correspond to the saturation zone of the device. The two most important parameters are $g_{m}$, transconductance gain, and $g_{ds}$, output conductance of the device which provides its output resistance [3,4]. The equations for those parameters are described as follows.

(9) $$g_{m}=\frac{di_{DS}}{dV_{GS}}{|}_{Q}=\sqrt{2\beta I_{D}\left(1+\lambda V_{DS}\right)}≅\sqrt{2\beta I_{DS}}$$

(10) $$g_{ds}=\frac{di_{DS}}{dV_{DS}}{|}_{Q}=\frac{\lambda I_{DS}}{1+\lambda V_{DS}}≅\lambda I_{DS}$$

From those expressions, λ is the channel modulation parameter shown in Table 2 and *β = k = μCox(W/L)* which was defined earlier for the large-signal model. These parameters are evaluated at the quiescent Q point where the device operates and the small-signal ignites. 

### 3.5. Parasitic Capacitances

The most important parasitic elements of the MOSFET are the capacitances due to the inherent field-effect operation of this transistor. The oxide and p-n junction capacitances are responsible for limitations of the frequency response which generates poles and zeros which are frequently analyzed to determine the stability of the device and the dominant bandwidth of the system. Three capacitances are considered in the conceptual design modeling:

i. “Oxide capacitance” is formed by the SiO_2_ between the gate terminal and the channel formed from drain to source. This capacitance is given by the gate oxide capacitance as follows.
  (11) $$C_{g}=WLC_{ox}=WL\epsilon_{ox}/t_{ox}$$
  where ε_ox_ is the oxide dielectric constant and *t_ox_* is the oxide thickness.
ii. “P-N junction capacitances” due to the reverse bias of the p-n junctions formed between drain/source to the substrate/bulk terminals of the device. This capacitance is evaluated using the built-in potential *φ_B_*, built-in zero-bias junction capacitance *C_j_*_0_, and the built-in junction capacitance *C_j_* as follows.
  (12) $$\varphi_{B}=V_{t}ln\left(\frac{N_{A}N_{D}}{n_{i}^{2}}\right)$$
  (13) $$C_{j0}=\sqrt{\frac{\epsilon_{Si}qN_{A}}{2\varphi_{B}}}$$
  (14) $$C_{j}=\frac{C_{j0}}{{\left(1-\frac{V_{j}}{\varphi_{B}}\right)}^{m}}$$
  where *n_i_* is the intrinsic carrier concentration in silicon, *N_A_/N_D_* are numbers of acceptor/donor atom concentrations per volume in material, *ε_Si_* is the silicon dielectric constant, and *V_j_* is the reverse bias voltage applied.

### 3.6. Passive Components

In microelectronic conceptual design, the passive components provide additional versatility in the consolidation and refinement of many integrated circuits that require compensation, parasitic element adjustment, antenna integration, and other possible maneuvers. For the conceptual modeling using the Excel methodologies, this paper considers only resistor and capacitor implementations. To implement resistances, Table 3 shows the values for sheet and contact resistances in the C5 ON-SEMI process [15] described by wafer runs from the corresponding foundry.

From Table 3, N+ and P+ are the n-type and p-type doped active silicon materials, respectively; POLY, POLY2, and POLY2_HR are polysilicon, polysilicon-2, and polysilicon-2-high-resistivity materials, respectively; N_WELL_ corresponds to the n-type well (where P-Type transistors are located); M1, M2, and M3 are metal-1, metal-2, and metal-3 layers, respectively, where the component connections are implemented inside the chip.

To implement capacitances, Table 4 shows the values for area, fringe and overlap capacitances in C5 ON-SEMI process [15]. The area capacitances for substrate, N+ active, P+ active, POLY, POLY2, M1 and M2 have units of aF/μ^2^, while the fringe and overlap capacitances are given in units of aF/μ. Parameters from table IV are very important in the compensation of analog integrated circuits that are used in instrumentation channels for processing signals coming from sensors and transducers. 

The equations to implement passive components are given as follows. Resistances made in N_well_, N+, P+, POLY, POLY2 and POLY2_HR:

(15) $$R_{T}\;=\;\left[Sheet\;Resistance\;Parameter\right]\;\times \;L/W$$

Capacitances made by POLY-POLY or POLY-Metal:

(16) $$C_{T}\;=\;\left[Capacitance\;Parameter\right]\;\times \;WL$$

Now that the model equations have been presented, the following sections will discuss the methodology used to describe the conceptual model for the CMOS microelectronic circuit. 

## 4. Excel Methods for CMOS Design

Conceptual design involves the use of parameters and constants to compute and size values of components and electrical variables [16,17]. However, sometimes the number of specifications given is larger than the number of degrees of freedom available to the designer trying to comply and fulfill them [3,4,27,28,29]. Therefore, in the design process, a series of decision points is necessary to iterate the processing flow before obtaining the final solution. Several trials are necessary and sometimes, intermediate simulations are required to analyze different alternative solutions. In this case, the use of Excel methods is convenient while advancing in the design process. There are three sorts of recommended methods:

- Single straight processing.
- Tabular straight processing.
- Two-dimensional processing.

### 4.1. Single Straight Processing Excel Method

**Figure 4:**
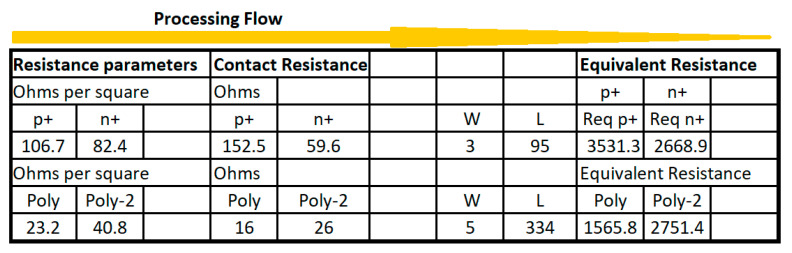
Single straight method to design CMOS resistances.

**Figure 5:**
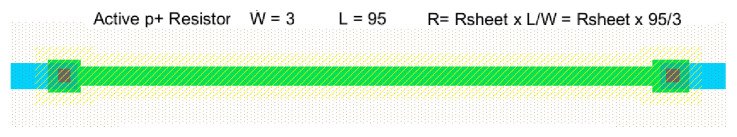
CMOS IC layout active p+ resistor design, 3.5 K, from Electric-VLSI.

**Figure 6:**
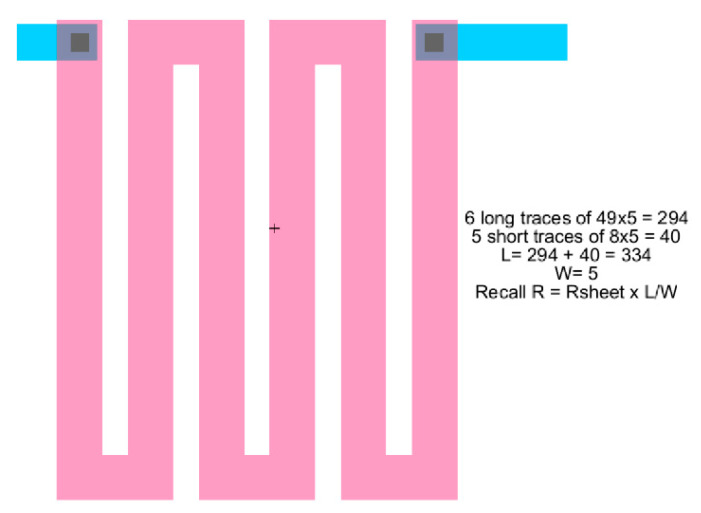
CMOS IC layout polysilicon resistor, 1.5 K, from Electric-VLSI.

In the single straight processing, the method proceeds in a horizontal fashion with the CMOS technology parameters in the first block of columns. Then, the processing flow continues sequentially, column by column, until the final desired calculation values are obtained. If several conditions are considered, then a row repetition is developed accordingly. Figure 4 shows single straight processing to design resistances using active p+, active n+, polysilicon and polysilicon-2 CMOS elements with their respective contact resistances and varying W and L sizes. Figure 5 and Figure 6 show the CMOS 500 nm IC layouts of 3.5 K and 1.5 K resistors, obtained from Electric-VLSI. The artwork was developed once the Excel method was used to calculate the number of sheets or squares required by the conceptual design. 

### 4.2. Tabular Straight Processing Excel Method

**Figure 7:**
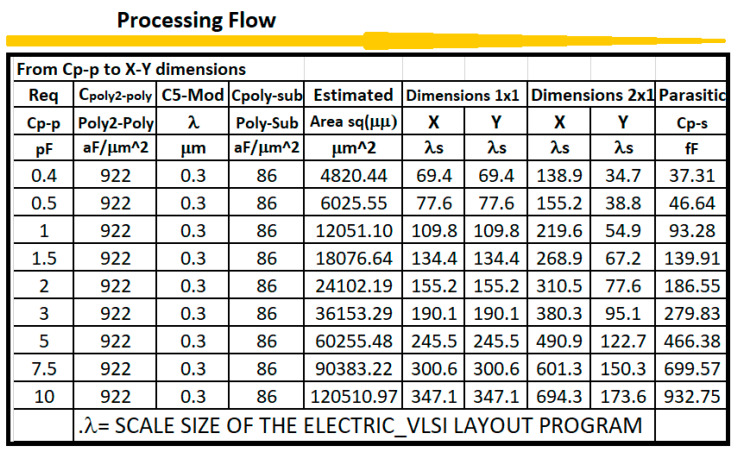
Tabular method to design CMOS capacitances. This Excel goes from capacitance value to X-Y dimensions.

**Figure 8:**
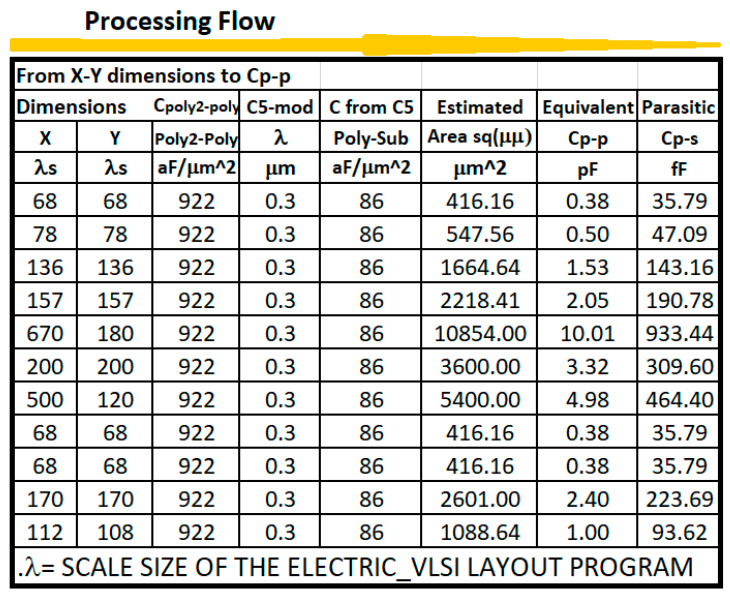
Tabular method to design CMOS capacitances. This Excel sheet goes from X-Y dimensions to capacitance value.

**Figure 9:**
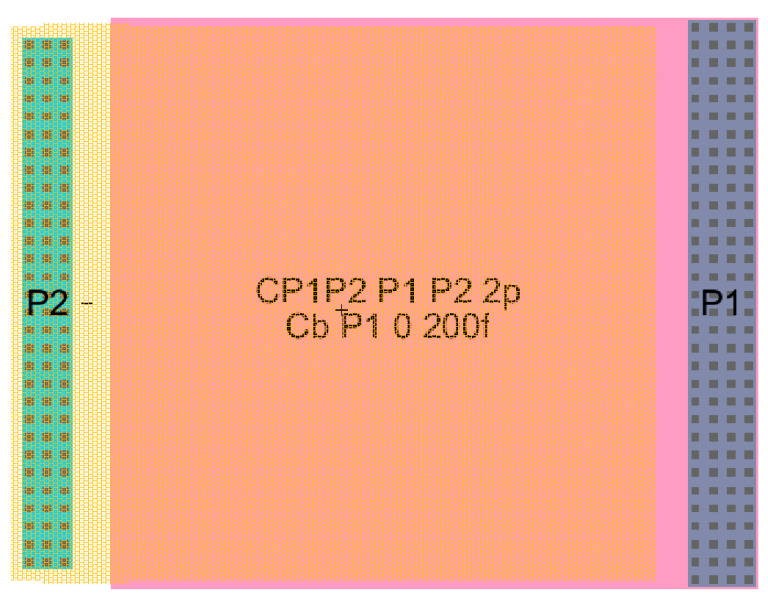
CMOS IC poly-poly 2 pF capacitance with squared layout from Electric-VLSI.

**Figure 10:**
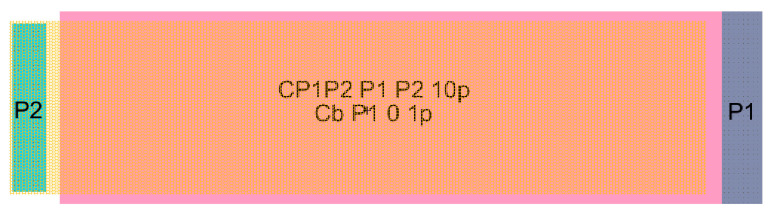
CMOS IC poly-poly 10 pF capacitance with rectangular X-Y layout from Electric-VLSI.

In the tabular processing, the method proceeds as usual, in a horizontal fashion to evaluate the design instance with the CMOS technology parameters in the first block of columns. However, this evaluation is repeated for a multiple number of instances while varying one or two specifications or parameters. Then, the processing flow continues sequentially, row by row, until the final desired instance is terminated. Each instance considered will be evaluated over a single row. Figure 7 and Figure 8 show tabular processing to design capacitances using poly-poly-2 CMOS 500 nm technology. Figure 5 starts with the value of the capacitance and ends up with squared plates in Poly-Poly to design the capacitance. Figure 7 starts with L/2 feature size (300 nm in this case) X-Y rectangular dimensions to obtain the effective capacitance value. Figure 9 and Figure 10 show the CMOS 500 nm IC layouts of 2 pF and 10 pF capacitances, obtained from Electric-VLSI. The artwork was also developed once the Excel methodology was used to evaluate the conceptual design. Capacitors from Figure 8 and Figure 9 are used for operational transconductance amplifier (OTA) compensation later in this paper. 

### 4.3. Two-Dimensional Processing Excel Method

**Figure 11:**
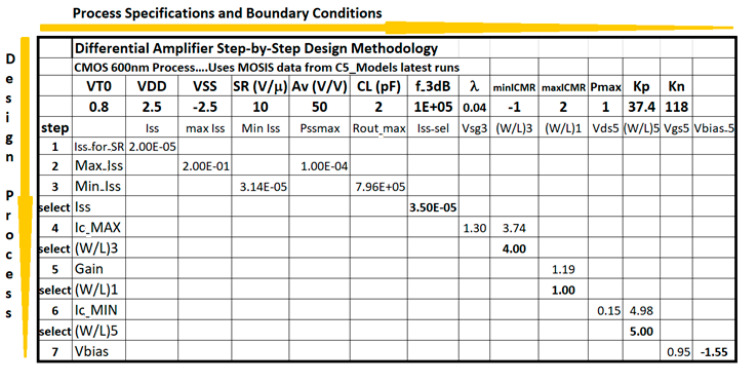
Two-dimensional processing method to design a differential amplifier. From the initial cell (top-left), the conceptual design progress downward, step by step, and to the right to size each transistor.

In two-dimensional processing, the method proceeds with the horizontal first row having the specifications and CMOS technology parameters of the conceptual design. Each row will define a step in the processing design flow such that the Excel will progress down and away from the first cell of the spreadsheet. The evaluations are arranged such that intermediate calculations follow a slope down from the early decisions all the way to the last decision. Usually, an iterative process is necessary to comply with two or three specifications with a single degree of freedom. For instance, with the bias current, I_SS_, of a differential amplifier, can fulfill expectations for Slew Rate (SR), Output Resistance (R_out_), and Power dissipation (P_diss_) in the conceptual design of a CMOS differential amplifier. Figure 11 illustrates the Excel method to develop the conceptual design for a CMOS differential amplifier. The method illustrates the step-by-step evaluation and decision-making process downward, and the CMOS technology specifications are shown rightwards as shown in Figure 11. The CMOS Technology characteristics flow horizontally to the right and the CMOS design equations, from (5) to (10), flow downwards illustrating the step-by-by step procedure. The evaluation of CMOS transistor size, W/L, goes along to fulfill the required specification. However, if a particular spec does not convince the design engineer, the processing flow can be stopped, and a recalculation with a different spec or different decision making is readily possible at every row. The processing flow continues row/column by row/column until the final step and size selection is terminated.

**Figure 12:**
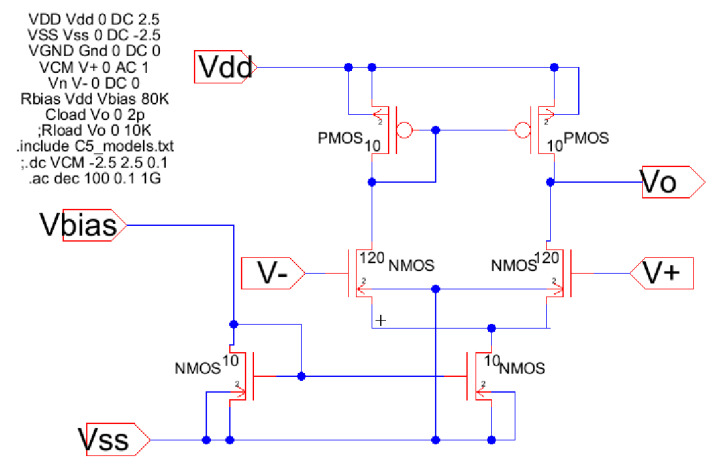
A differential amplifier schematic using CMOS 500 nm technology.

**Figure 13:**
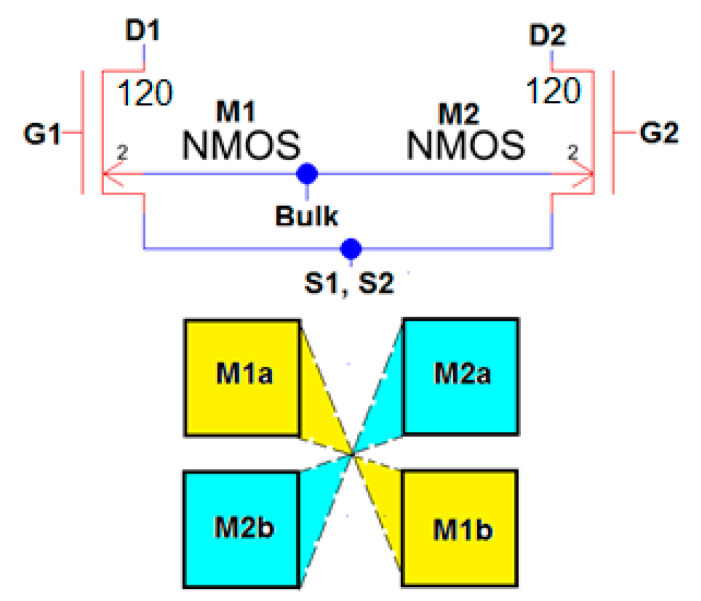
Common centroid layout recommended for big matched differential pair transistors [3,4].

Figure 12 shows the schematic from Electric-VLSI of a differential amplifier using results from the Excel two-dimensional method. In this case, every value sizing the MOSFETs means L/2 times or half the feature size of the technology. For instance, the middle twin transistors have a size of W = 120 × (0.5/2) = 30 μm by L = 2 × (0.5/2) = 0.5 μm. Furthermore, the SPICE code to perform DC and AC testing in this conceptual design of the device is shown in Figure 12. Once this schematic circuit model is tested, the next step is developing the layout. Differential amplifiers are the core of every instrumentation amplifier and their layout must be considered very carefully. Figure 13 shows the strategy recommended by J. Baker [4] to develop a common centroid layout.

**Figure 14:**
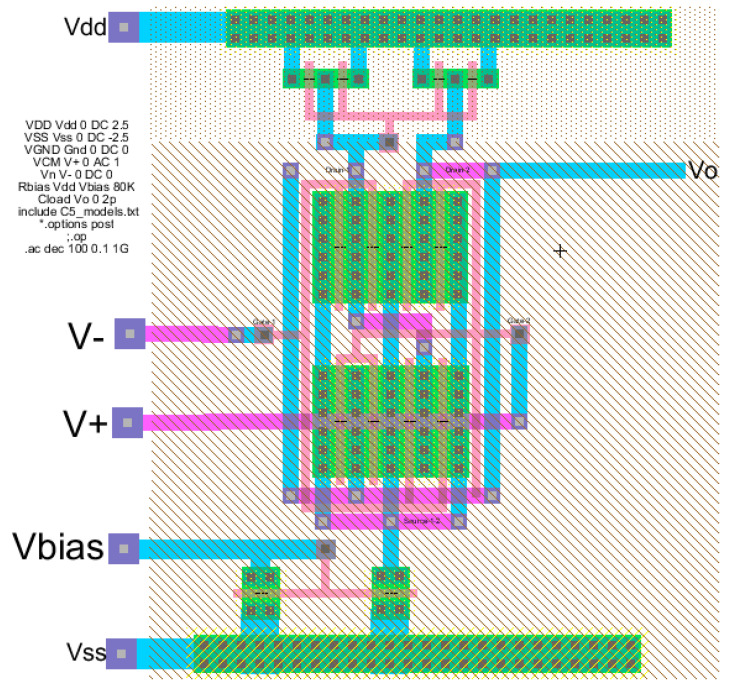
Conceptual design layout (using Electric-VLSI) of a differential amplifier from an Excel method.

With careful development by considering the DRC (direct rule checking) from Electric-VLSI CMOS 500 nm kit, the layout shown in Figure 14 is developed by using the evaluations and conceptual model obtained from the Excel two-dimensional method.

## 5. Methodologies for Complete Amplifier Design

The Excel methodologies shown previously can be applied to develop conceptual designs of complete functional blocks such as a cascode amplifier with its bias circuit, and a two-stage operational transconductance amplifiers (OTA). The OTA amplifier includes a compensation capacitance which is necessary to ensure stability and reliable operation for the required frequency response. Even though the following design examples are not very specialized, the literature shows many examples of more specialized conceptual designs where microelectronic design has been extended [30,31,32,33]. The design flow that students must follow to perform the conceptual design of the microelectronic device is given as follows:

1. Select or create the basic structure. This step consists of obtaining the schematic showing the transistors and their connections. This diagram does not change through the design process unless the specs cannot be met.
2. Set up the Excel spreadsheet to show the specifications and boundary conditions in the first line. Make sure that the units are consistent all the way through the design process. Develop the decision process line by line using the equations and specs. The design flow goes downwards.
3. Select the DC currents and transistor sizes to meet specs. This is where the major design effort will go. Simulators are used to aid the designer in this phase. However, a rough performance of the circuit should be known a priori.
4. Physical implementation. Layout of the transistors, floor-planning the connections, pinouts, power supply buses, and grounds. Extraction of physical parasitic and re-simulation. Verify that the layout is a physical representation of the circuit.
5. Furthermore, in designing a multi-stage amplifier where two or three stages integrate the complete device, the following considerations are made:
  - The characterization of the microelectronic device is the initial fundamental step in the analysis and design of the multi-stage amplifier.
  - Ideal analysis provides the first insight to the circuit operation by performing circuit analysis over the external components.
  - Practical models of the op-amp include static and dynamic parameters.
  - The Excel method helps in developing the overall step-by-step procedure which accounts for all the requirements and specifications
  - The design of multi-stage devices involves the following boundary conditions and requirements:
    - Boundary conditions: CMOS technology, process specs (*V_T0_, C_ox_, K’*), supply voltage and current range (*V_DD_, V_SS_, I_SS_*), operating temperature (*T_o_*) and range.
    - Requirements: Gain (*A_v_*, *A_i_*), gain bandwidth (*GB*), settling time (*T_s_*), slew rate (*SR*), input common mode range (*ICMR*), common mode rejection ratio (CMRR), power supply rejection ratio (*PSRR*), output voltage swing *(v_out_ (max), v_out_ (min)*), output resistance (*R_out_*), offset voltage (*V_OS_*), noise (*e_out_ ^2^*), layout area.
    - Verify that intermediate stages couple correctly without causing instabilities such as the influence of mirror poles in the transfer function. The Excel method can be used to revise and iterate the process in search of a robust device.
  - Compensation techniques involve: Miller, feed-forward, and self-compensation schemes.

### 5.1. Cascode Amplifier with Bias Source

**Figure 15:**
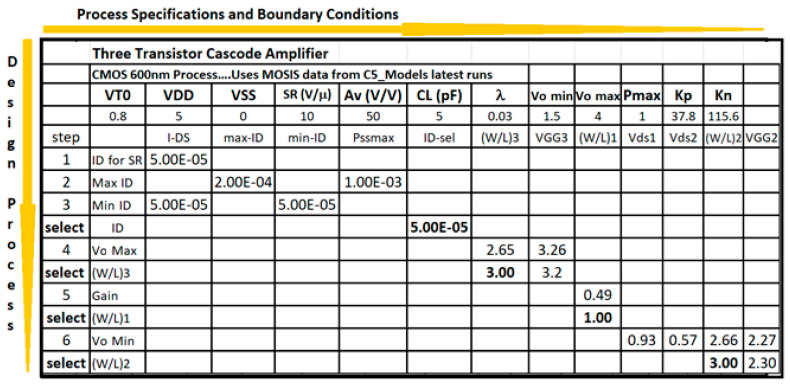
Two-dimensional processing method to design a CMOS cascode amplifier.

The cascode amplifier obtains a higher gain and output resistance than the traditional inverting amplifier stages. Typical design parameters are slew rate (SR), output swing, and power dissipation for a simple cascode stage. Figure 15 illustrates the Excel method to develop the conceptual design for a cascode amplifier. As mentioned before, the method illustrates the step-by-step evaluation and decision-making process downwards and the processing flow goes rightwards. Again, the CMOS Technology characteristics and specifications flow horizontally to the right and the CMOS design equations, from (5) to (10), flow downwards illustrating the step-by-by step procedure. The evaluation of CMOS transistor size, W/L, goes along to fulfill the required specification.

**Figure 16:**
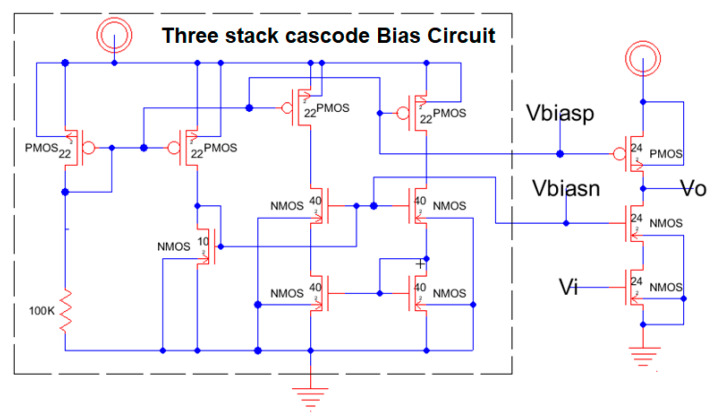
A three stack cascode amplifier schematic using CMOS 500 nm technology.

Figure 16 shows the schematic from Electric-VLSI of the conceptual design of the cascode amplifier which includes the bias network on the left of the stacked three MOSFET from the right. The bias circuit was implemented and evaluated in a separate analysis using the guidelines from J. Baker [4]. Once this schematic circuit model is tested, the next step is to develop the layout.

**Figure 17:**
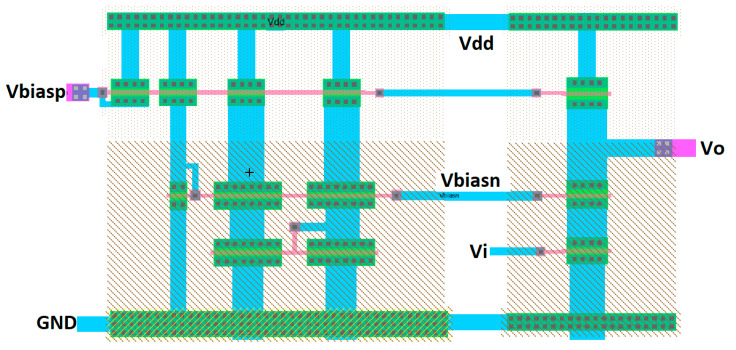
Conceptual design layout (using Electric-VLSI) of a three stage cascode from an Excel method.

The layout is developed using Electric-VLSI and includes both the bias circuit and the three stacked transistors as shown previously in Figure 16. Figure 17 illustrates the layout design with the corresponding V_dd_ = 5 V and Vss = 0 (ground). This layout shows the transistors in horizontal layouts where the lower two levels include N-type transistors, and the upper level includes the P-type transistors which appear in the circuit schematic from Figure 16. The layout also shows the p-well and n-well over the lower two N-channel transistor levels and the upper P-channel transistor level, respectively. 

### 5.2. OTA with Miller Compensation

**Figure 18:**
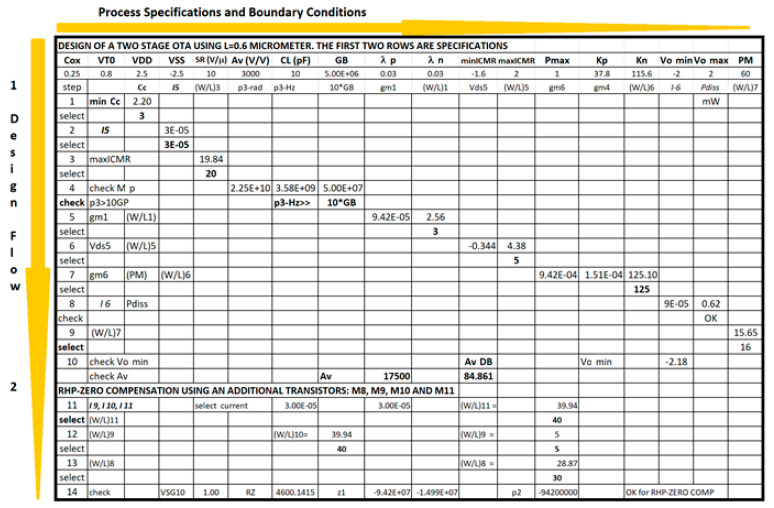
Conceptual OTA design with two downward decision flows.

The development of a conceptual model for an operational transconductance amplifier with Miller compensation has an additional complexity of calculating the feedback capacitance that operates the amplifier in a stable and reliable regime. The phase margin PM > 50° and the sizing of more than 10 transistors make the Excel method larger. Figure 18 shows the Excel spreadsheet workout with the evaluation of transistor sizes and dominant pole calculations. 

**Figure 19:**
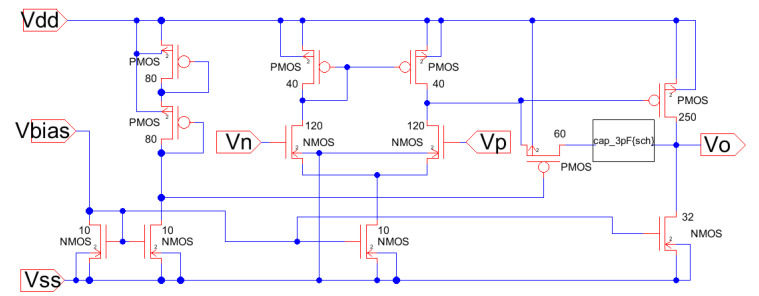
Electric-VLSI schematic of the CMOS OTA with RHP-zero compensation.

**Figure 20:**
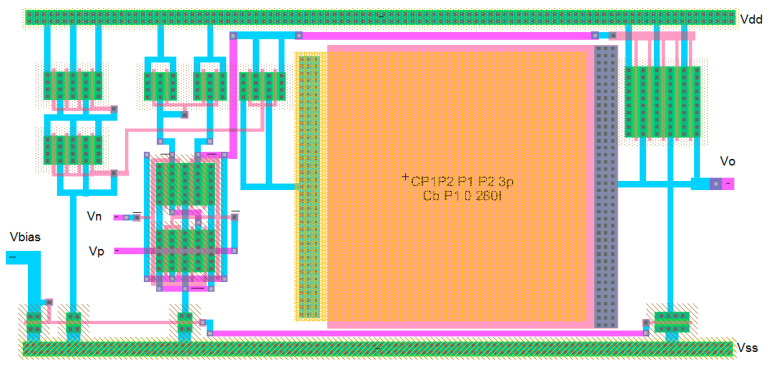
Electric-VLSI layout of the CMOS OTA with RHP-zero compensation.

Again, the procedure shows the conceptual design strategy mentioned before, the CMOS technology characteristics and specifications flow horizontally to the right and the CMOS design equations, from (5) to (10), flows downwards illustrating the step-by-by step procedure. In this case, Figure 18 has two downward decision flows. The first one evaluates the design with the sole calculation of the Miller compensation capacitance to provide the required phase margin and stability criteria [3,4,17,27]. The second design decision flow determines the size of a transistor to locate a right-hand side pole (RHP) exactly to cancel the second pole. This way the dominant pole will be extremely alone well inside the gain bandwidth and the amplifier will have, even higher, phase margin PM. Figure 19 shows the Electric-VLSI layout schematic of the conceptual design obtained for the 500 nm CMOS technology. Figure 20 illustrates the layout of the OTA amplifier with the area dominance of the compensation capacitance C_c_ = 3 pF. Those capacitances are extremely large and, in this case, the layout generated has a squared shape, like the ones developed with a single straight Excel methodology for capacitors. The layout shows also the common centroid differential amplifier stage developed before and the big, 125 μm/1 μm, p-channel MOSFET transistor right above the output voltage of the amplifier.

### 5.3. Performance Simulation Tests

The Excel methods are used to synthesize the conceptual designs of integrated circuits and devices to teach and develop successful strategies that can be repeated for different CMOS technologies. This section will compare the expected specifications defined initially with the schematic and layout results from SPICE simulations run by Electric-VLSI using the LTSpice program as a kernel.

Four conceptual design cases are analyzed which are part of a formal course in microelectronics [28]. The design problems are:

1. Differential amplifier using common centroid layout.
2. The three-stack MOSFET cascode amplifier using the corresponding bias power reference.
3. The two-stage OTA stage with Miller compensation and having the RHP cancel the second dominant pole.
4. A three-stage op-amp using shunt feedback output stage to enhance the output resistance.

The differential amplifier results are summarized in Table 5. This amplifier stage is shown in Figure 11 and Figure 13 and resolved using the two-dimensional processing method from the Excel methodology illustrated in Figure 10. The results comply with all the specifications established for the conceptual model using the CMOS 500 nm technology as illustrated in Table 5. Some parameters differ slightly because of the iterative nature of the design decision flow in which the number of requirements is higher than the number of degrees of freedom available: three transistor sizes (W/L) and the operating Q point of the current-sinking at the lower transistor in Figure 13. 

Table 6 illustrates the results for the cascode amplifier conceptual model developed using the Excel method shown in Figure 14. Again, this amplifier stage, shown in Figure 15 and Figure 16, describes the fulfillment of all the specifications established for the conceptual model using the CMOS 500 nm technology. The power dissipation shown for this amplifier includes the three stack of transistors and the bias reference network shown in Figure 15 and Figure 16. Again the number of requirements is higher than the number of degrees of freedom available: three transistor sizes (W/L) and the operating Q point of the current-sinking at the lower transistor in Figure 16. The two previous amplifiers, differential, and cascode are not used as independent amplifiers, but they are part of a larger multistage amplifier or microelectronics functional block. Therefore, the conceptual models for a highly specialized microelectronic device contain 2, 3, or more of those primitive amplifiers described previously. Now we will present results for the conceptual model of a two-stage OTA and of a three-stage operational amplifier. Table 7 shows results for the conceptual model of a two-stage OTA that includes a differential amplifier and an inverting amplifier that drives the load. This was the amplifier illustrated by Figure 18 and Figure 19 and developed using the methodology of Figure 17. This OTA conceptual design includes additional specifications such as offset voltage, output swing, phase margin, power supply rejection ratio (PSRR), gain-bandwidth GB, and settling time. This is a preview of the project that the students in the microelectronics course develop at the end of the semester.

Finally, Table 8 illustrates the results obtained from a conceptual design of a 3-stage op-amp using an additional third stage with a shunt feedback scheme to reduce the output resistance of the device. The results are good with a low value with the negative slew-rate (SR) of −9 V/μs which needs to be improved from this conceptual design developed using the methodologies discussed in this paper. 

**Figure 21:**
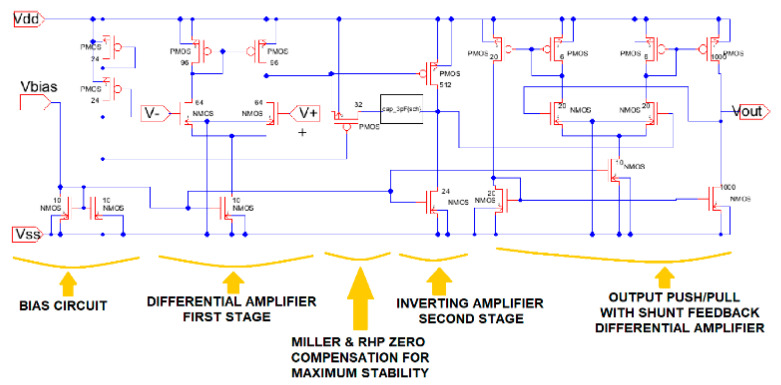
Electric-VLSI schematic of the 3-stage CMOS op-amp with RHP-zero compensation.

The schematic of the three-stage op-amp conceptual design is shown in Figure 21. This figure illustrates the main sub-components of the device: bias and reference voltage circuit, first stage differential amplifier, Miller and right-hand plane zero (RHP) compensation for maximum stability, inverting amplifier second stage, and the output push/pull with shunt feedback differential amplifier that provides a lower output resistance in the device.

The conceptual design developed in this paper shows one of the major steps in designing analog integrated circuits (IC) for electronic instrumentation devices required by electronics, biomedical, robotics, and computer engineering majors. In analog IC design, a good combination of function or application with IC technology is necessary to obtain a successful solution. The Excel methodologies are an additional tool to validate and verify the conceptual model required before the device is sent to the foundry facility. Analog IC design consists of three major steps [3]: electric design, physical design (layout), and test design (testing). Engineers and designers must be flexible, use techniques such as the Excel methods, and have a skill set that allows them to simplify and understand a complex conceptual design problem. In microelectronics, device IC design is driven by improving technologies rather than new technologies [3]. The engineer should be aware that sometimes analog systems applications, where speed, area, or power, have certain advantages over the digital approach. Even using Excel methods, deep-submicron (DSM) technologies offer great challenges to the creativity of engineers and designers of IC microelectronic devices.

## 6. Analysis of Results

To further increase the potential of using Excel methods in developing instrumentation amplifiers for biomedical applications, we examined a project case study using the methodology to design a 3-stage amplifier having a low voltage and low power operation in the strong inversion zone. Undergraduate students during the spring semester of 2021 in the microelectronics course at Tecnológico de Monterrey developed a project where they used the methods learned in class [34]. This design was going to be used as a subsystem of a bioinstrumentation amplifier required in sensor signal conditioning applications. 

**Figure 22:**
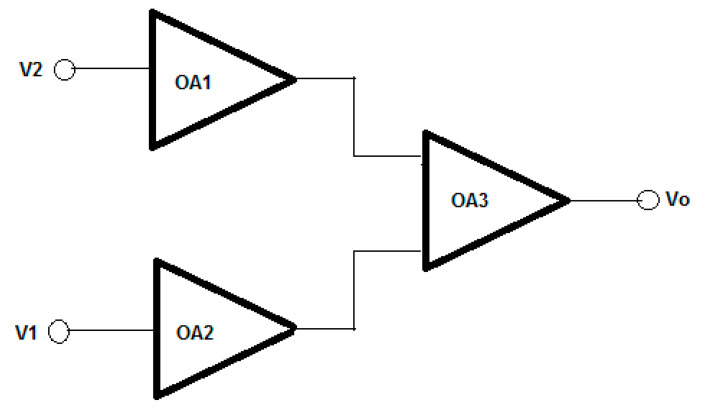
Classic topology for the instrumentation amplifier used in biomedical applications.

The project consisted of the development of a three-element instrumentation amplifier for biomedical applications. Figure 22 illustrates the basic scheme where two op-amps receive the differential mode input signals, and a third op-amp changes the signal from differential mode to single-ended mode referenced to ground.

In Figure 22, each op-amp (OA1, OA2, and OA3) must be selected from possible topologies seen in class to achieve certain performance characteristics. Those op-amps have the following components:

1. First stage differential amplifier;
2. Second stage common source amplifier;
3. Third stage push-pull output stage;
4. Power source to provide the required bias currents and voltages.

**Figure 23:**
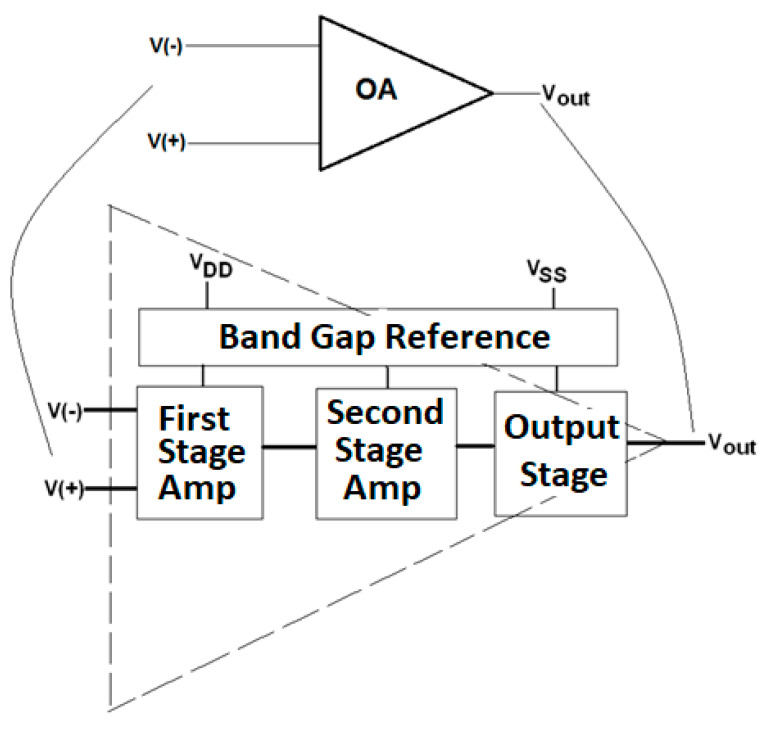
Overall block diagram of each operational amplifier.

Figure 23 shows a block diagram of each op-amp with all the functional parts of the system. For the power source, the students have the option of developing a high-performance bandgap reference source that is stable with respect to variations in supply voltage, temperature, and noise. 

The requirements and specifications provided in Table 9 are to be met for each of the op-amp blocks.

The students began with a literature survey before selecting the device to develop from the conceptual requirements, theoretical development, schematic development, and layout implementation using Electric_VLSI [35,36,37,38]. Instead of adding a third stage at the output of the two-stage OTA device, they added a second stage differential-amplifier with compensation between the first differential stage and the output stage. This results in improving the frequency response and sacrificing some gain and having a better phase margin for even robust design. Even with this modification, the simulations show an increase in the overall gain compared to the last three stage designs (Section 5). The compensation capacitor *Cc* and *Rz* are kept for both differential stages, and the output terminals of both compensation elements are connected all the way to the output of the circuit. The methodology generates the transistor dimensions and capacitor values illustrated in Table 10 and Table 11.

Considering the transistor dimensions for this new three stage amplifier design, the first cut expected parameters are calculated as follows, assuming long channel models.

1. Slew rate.

(17) $$SR=\frac{I_{5}}{C_{c}}=\frac{10\mu }{0.5p}=20\;V/\mu s$$

2. Estimated amplifier gain.

(18) $$A_{v0}={\left(\frac{g_{m2}}{g_{m2}+g_{m4}}\right)}^{2}\cdot \frac{g_{m6}}{g_{ds6}+g_{ds7}}={\left(\frac{g_{m2}}{I_{5}/2\left(\lambda_{2}+\lambda_{4}\right)}\right)}^{2}\cdot \frac{g_{m6}}{I_{6}(\lambda_{6}+\lambda_{7})}$$

(19) $$g_{m2}=\sqrt{2\beta_{2}I_{2}}=\sqrt{2\times 115.6\mu \times 8\times 5\mu }=96.17\;\mu S$$

(20) $$g_{m6}=942.5\;\mu S$$

(21) $$A_{v0}=={\left(\frac{96.16\mu }{5\mu \left(0.04+0.05\right)}\right)}^{2}\cdot \frac{942.5\mu }{92.14\mu \left(0.04+0.05\right)}=134.3\;\;\mathrm{dB}$$

3. Power dissipation.

(22) $$P_{diss}\;=\;\left(2I_{5}+I_{7}\right)\left(V_{dd}+\left|V_{ss}\right|\right)\;=\;\left(2\times 10\mu +92.14\mu \right)\left(2\times 1.8\right)\;=\;403.7\;\mu W$$

4. Output swing.

(23) $$V_{out}\left(min\right)=V_{ss}-V_{DS7}\left(sat\right)=-1.8+\sqrt{\frac{2I_{7}}{k_{N}´{\left(W/L\right)}_{7}}}=1.8+\sqrt{\frac{2\times 92.14\mu }{115.6\mu \times 22.5}}=-1.53\;V$$

(24) $$V_{out}\left(max\right)=V_{dd}-V_{SD6}\left(sat\right)=1.8-\sqrt{\frac{2I_{6}}{k_{P}´{\left(W/L\right)}_{6}}}=\sqrt{\frac{2\times 92.14\mu }{37.8\mu \times 130}}=1.607$$

5. Input common mode range, ICMR.

(25) $${\left(W/L\right)}_{5}=\frac{2I_{5}}{k_{N}´V_{DS5}{\left(sat\right)}^{2}}=\frac{2\times 10\mu }{k_{N}´{\left(-V_{ss}+ICMR^{-}-\sqrt{\frac{I_{5}}{\beta_{1}}}-V_{T1}\left(max\right)\right)}^{2}}$$

(26) $$ICMR^{-}=-0.93V\;$$

(27) $${\left(W/L\right)}_{3}=\frac{I_{5}}{k_{P}´{\left(V_{dd}+ICMR^{+}-|V_{T3}|\left(max\right)+V_{T1}\left(min\right)\right)}^{2}}$$

(28) $$ICMR^{+}=1.69V$$

6. Output resistance.

(29) $$R_{out}=\frac{1}{g_{ds6}+g_{ds7}}=\frac{1}{I_{6}(\lambda_{N}+\lambda_{P})}=\frac{1}{92.14\mu \left(0.04+0.05\right)}=120.5\;K\Omega$$

**Figure 24:**
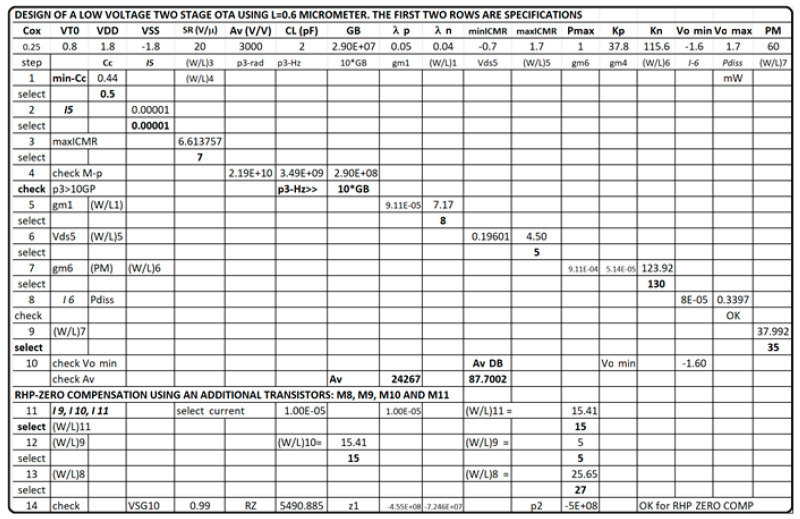
Excel methodology for the first part two-stage OTA conceptual design, before connecting the third stage to achieve higher gain and robust conceptual design.

Afterward, the circuit schematic and layout are developed in Electric VLSI to perform simulations tests. The compensation capacitor is designed as a poly-poly2 capacitor. The area for the capacitor is 78 by 78 lambda, with a 47 fF parasitic capacitance. Figure 24 shows the Excel first cut approximation used for the first part of the conceptual design. 

**Figure 25:**
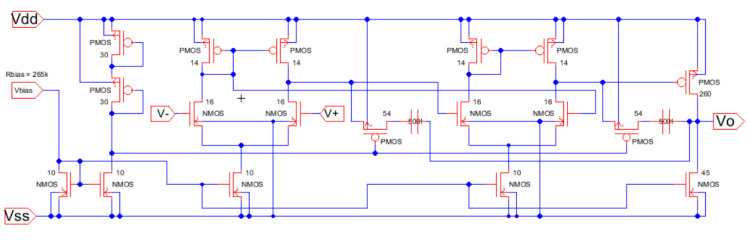
Schematic diagram from Electric_VLSI for the three-stage low-power high-gain operational amplifier.

**Figure 26:**
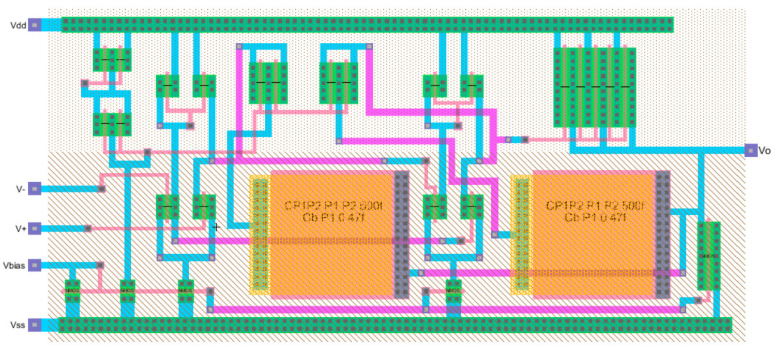
Layout diagram from Electric_VLSI for the three-stage low-power high-gain operational amplifier.

The Excel design spreadsheet shown in Figure 24 replicates the strategy which has been proposed in this article. The CMOS technology characteristics and specifications flow horizontally to the right and the CMOS design equations, from (5) to (10), Table 1 and Table 2, flow downwards illustrating the step-by-by step procedure. Figure 25 and Figure 26 illustrate the schematic and layout diagrams for the three-stage amplifier designed using the methodology shown and initiating with the conceptual design equations coming from the long channel model. 

**Figure 27:**
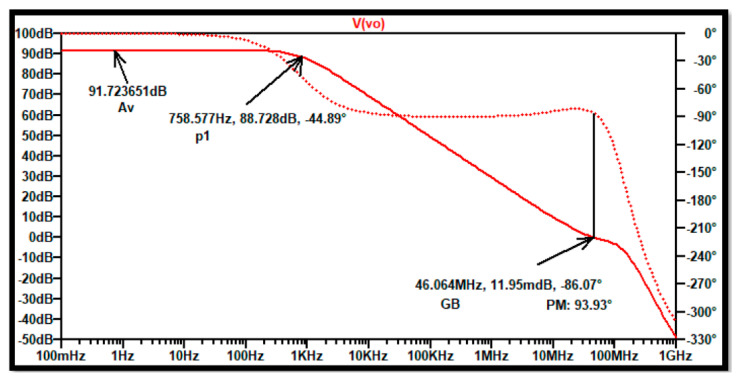
Frequency sweep using. AC analysis in SPICE for the three-stage low-power high-gain op-amp Layout.

The output simulation runs show results that comply with the major specifications required by the design. Figure 27 illustrates the layout frequency sweep where the dB gain, gain bandwidth, and the phase margin are displayed. The DC gain obtained is 91.7 dB, the gain bandwidth GB is 46 MH and the phase margin is now close to 94°, which makes a very robust device. 

**Figure 28:**
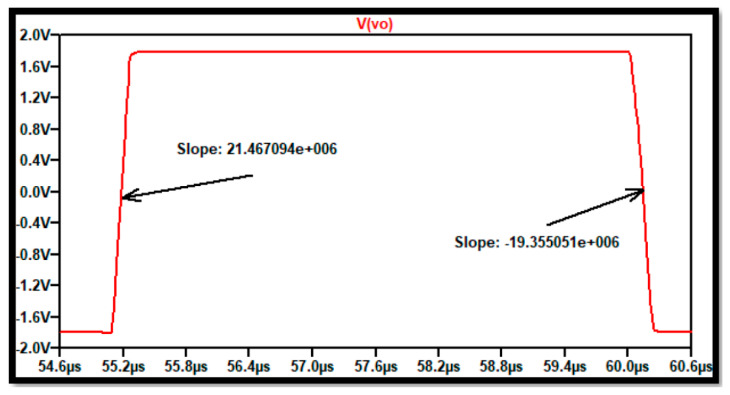
Time domain response using transient analysis in SPICE for the three-stage low-power high-gain op-amp layout.

Figure 28 shows the time domain simulation to verify the slew rate response. The graph shows $SR^{+}=21.47\;V/\mu s$ and $SR^{-}=-19.35\;V/\mu s$ which is much more than the required response. 

To summarize the complete results for all the theoretical and expected parameters, Table 12 compares:

1. Desired requirements for the conceptual design;
2. Theoretical calculations using the Excel method;
3. Simulation results of the schematic circuit;
4. Simulation results of the layout circuit.

The results compare well to the specified requirements except for the maximum positive swing of the output signal which does not reach the specified value of 1.4 V. Furthermore, the power dissipation is slightly higher than anticipated by the theoretical calculations of 0.403 mW. However, very good results are obtained in gain bandwidth (GB), CMRR, PSRR, phase margin PM, and noise. The requirements for slew rate (SR) and DC gain ($A_{v0}$) are barely achieved. Some of those specifications could have been obtained with additional calibration and refinements in the final conceptual design.

The student team [34] decided to compare the conceptual design of this low-power operational amplifier with one that appears in the literature with similar characteristics and is used in similar biomedical applications [35]. Table 13 shows this comparison where the supply voltage and the power consumption appear higher in our design. The load capacitance was a specification given by the instructor. Slew rate and bandwidth show a significantly higher performance in our design; however, those were parameters also required by the instructor. The chip area is twice in our design, however, in this case, the instructor did not have any restrictions here and no optimization was performed to reduce the layout artwork whatsoever. Table 13 results, however, illustrate comparisons under different specifications such as the load capacitance and voltage supply. Reference [35] used $C_{L}=30\;\mathrm{pF}$ and ±1.65 V, whereas our example used $C_{L}=2\;\mathrm{pF}$ and ±1.8 V, respectively. Some of those conditions were imposed by the course instructor. These changes produced large differences in closed-loop bandwidth and slew rate (SR) as seen by Table 11 results. Furthermore, the power dissipation and chip area obtained here are twice the values obtained by reference [35]. Another way to reduce the power dissipation and voltage supply could have been if they have designed the amplifier to operate in the subthreshold zone. However, the model equations in the Excel method would need to be changed to include the subthreshold model whatsoever. This comparison is an excellent way to encourage confidence in students about the design of integrated circuits at the nanoscale level [36]. 

**Figure 29:**
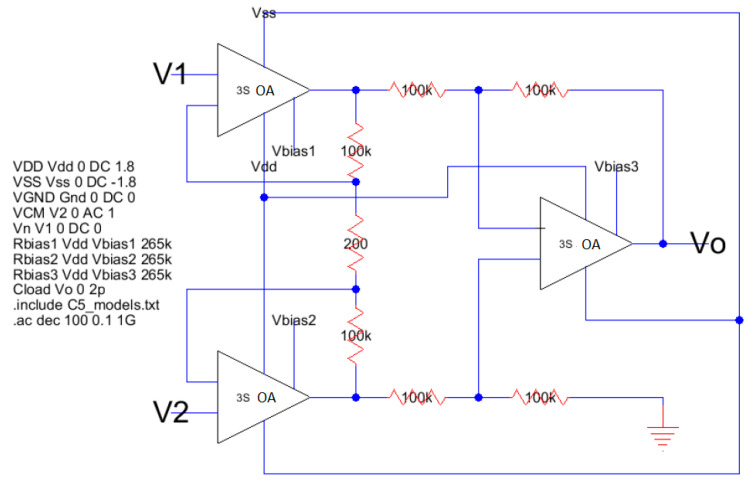
Instrumentation amplifier system simulation test using icon-view with Electric_VLSI.

A system simulation test was performed by students with an instrumentation amplifier working with a gain of 60 dB or 1000 *v/v* using the traditional three op-amp topology. Figure 29 shows the Icon-View of the simulation experiment having three op-amps (3S_OA) and the required resistors to provide the gain factor and the bias of the device. The figure shows on the left-hand side the SPICE code to run the frequency domain test (.ac dec 100 0.1 1 G). Furthermore, the MOSFET models command < include C5_models.txt> describes the 500 nm technology used in this case. 

**Figure 30:**
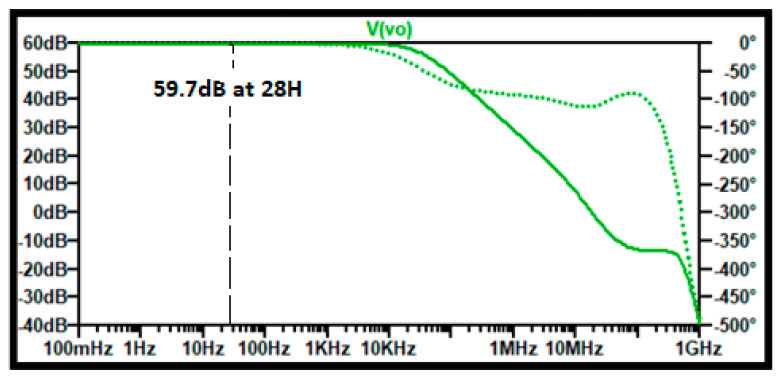
SPICE AC frequency domain test for the instrumentation amplifier.

The frequency-domain tests show a DC gain very close to the required for this instrumentation amplifier. Figure 30 shows the output voltage graph with a measured gain of 59.7 dB, approximately 966 V/V, which shows less than 5 % error with respect to the required 1000 V/V gain. This gain goes along to up to 10 KH in bandwidth. 

**Figure 31:**
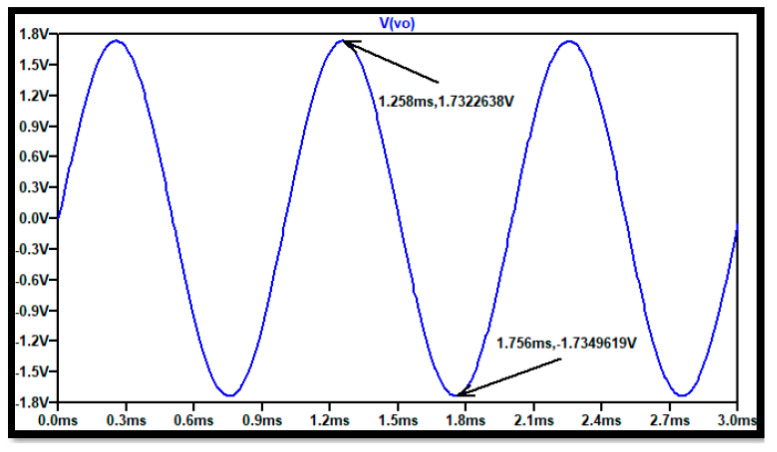
Time domain test for the instrumentation amplifier, where the maximum rail to rail swing is shown.

Finally, a time-domain system test for the instrumentation amplifier was developed to find the maximum output symmetrical swing for the device when amplifying the biomedical sensor signal. This transient test was performed with a 1.8 mV amplitude at 1000 H differential mode signal (V2-V1) at the input. Figure 31 shows the output signal with a+1.7323 V to −1.735 V swing which also shows the gain of 966 *v/v* for the instrumentation amplifier configuration. 

The designed operational amplifier and the instrumentational amplifier have good features for biomedical portable sensor conditioning applications. ECG and EEG biopotentials can be processed, and a good front device can be implemented using the conceptual design generated using these methods. The design of micro-power-operational amplifiers to fulfill biomedical instrumentation characteristics has become a huge yet complicated research task with great opportunity areas for improvement and innovation. The design proposed by the students offers further improvement opportunities (like reducing output resistance) yet possesses interesting features that would hopefully serve for its application in sensor signal conditioning in biomedical instrumentation.

**Figure 32:**
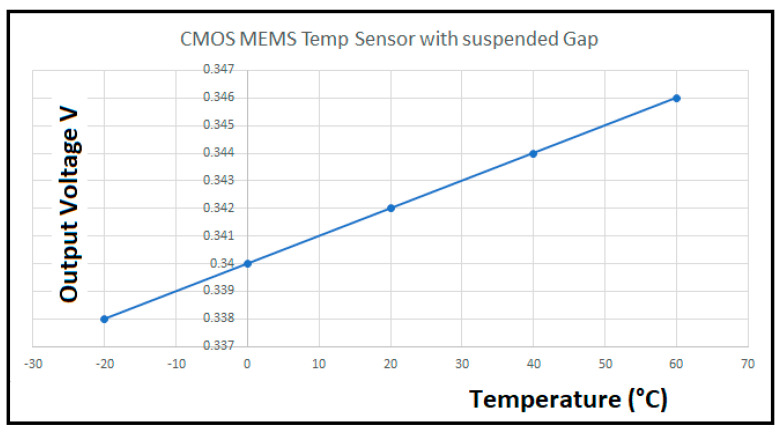
Output voltage for a CMOS MEMS temperature sensor with suspended gap from reference [42].

**Figure 33:**
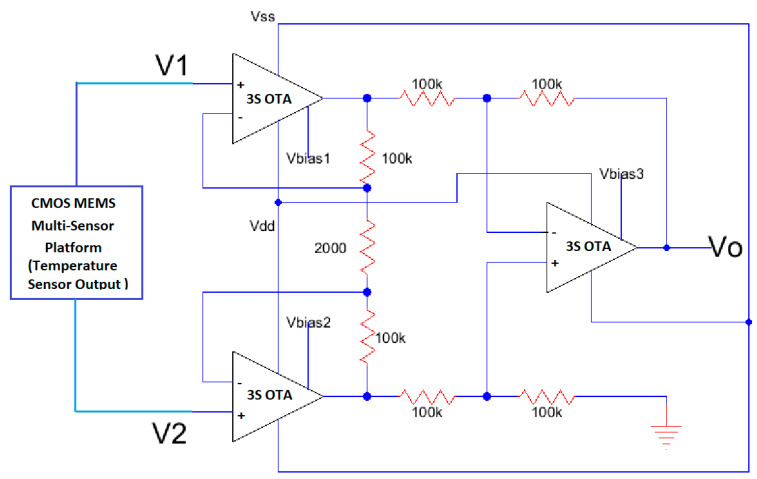
Instrumentation amplifier to condition the output from the temperature sensor described by the multiplatform of reference [42].

**Figure 34:**
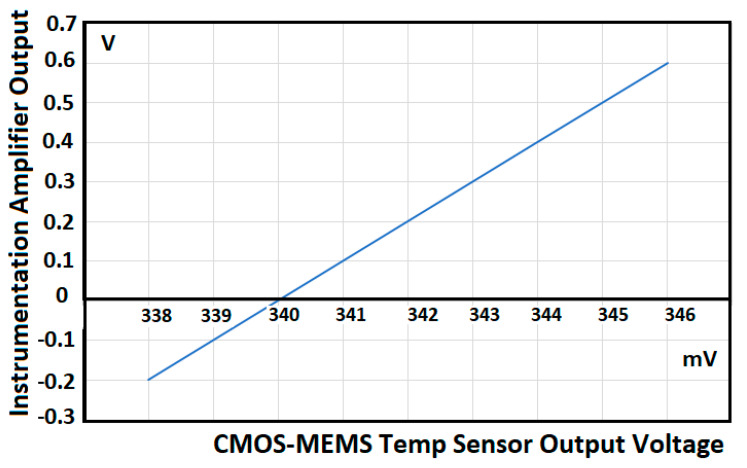
The linear signal conditioning performed by the instrumentation amplifier on signals coming from the CMOS-MEMS Temp Sensor Multiplatform [42].

Another application of the low power operational amplifier is to perform signal conditioning in signals coming from CMOS-MEMS sensors [39,40,41,42] where the CMOS amplifiers are used as the standard electronic system maneuvering platform. For instance, in [42] the low power operational amplifier is used as a high input impedance instrumentation amplifier to condition signals coming from a micro-hotplate in either Pirani, Temperature, or Gas CMOS-compatible MEMS sensors. Three operational amplifiers, designed in this paper, are set up as instrumentation amplifiers like the one shown in Figure 29. This configuration is ideal for this multifunctional sensor platform application, particularly for the results obtained by the temperature sensor as shown in Figure 32. Figure 33 shows the new instrumentation amplifier configuration used to condition the signal coming from the multiplatform temperature sensor [42] to obtain the results shown in Table 14. Figure 34 illustrates the linear conditioning performed by the instrumentation amplifier designed using the low power OTA developed by the students. 

The results obtained in this CMOS-MEMS sensor application confirm the possibility of using the EXCEL methods to perform first cut front end device design and testing. In teaching microelectronics, sometimes the use of high-performance software tools deviates attention toward the final objective and competence development for electronics engineers. The analysis of results from the previous two examples shows that the use of EXCEL methods provides a fertile background to start conceptual design processes without requiring complex or expensive software which is not readily available in many universities around the globe. Finally, to account for parasitic capacitances and temperature effects over the operating point of the amplifiers, Appendix A provides additional equations that can be added to the Excel method to find those fluctuations when using Spice simulations of the device. Appendix A.1 shows the parasitic capacitance modeling and Appendix A.2 illustrates the temperature model of this technology.

## 7. Conclusions

This paper described methods for developing the conceptual design of microelectronic circuits before performing schematic and layout simulations. The Excel methods developed here are useful for following guidelines and to make design decisions during iterative processes of designing where the number of specification requirements is larger than the degrees of freedom available to maneuver the design. The “thinking model” for paper and pencil calculations is described with the major roles and variants in the computation of electrical variables and sizes of the transistors that integrate analog microelectronic devices.

The Excel method may include straight single dimension, tabular, and two-dimensional methods that allow the design engineer to go step by step in the decision flow which navigates downwards in the Excel spreadsheet. Several examples of conceptual designs are shown with the corresponding spreadsheet diagram, circuit schematic, and layout design to perform simulation tests. The examples include passive components: resistors and capacitors, functional subcircuits such as primitive amplifiers, complete specialized amplifiers such as OTA devices, and term projects where students research specialized devices for biomedical instrumentation applications. The full-blown methodology includes the Excel method, schematic development, layout implementation, and simulation test, and it is used for conceptual design development in microelectronics courses at undergraduate and graduate levels. The undergraduate courses include industrial partner participation to develop instrumentation systems specified by industrial needs [43]; as a result, these educational collaborations with other entities have been applied in our academic educational practices and/or by applying our new educational model TEC21 at the undergraduate level [44,45,46,47]. As mentioned earlier, the main contributions of this research are in teaching CMOS microelectronics in which the method helps the instruction of the following points:

(1) Conceptual design evaluations using the traditional equations and preparation of layout implementation by setting up the schematic of the design.
(2) Providing possible design solutions, with layouts developed and comparing results with the thinking model specs to validate the third phase of the microelectronics design process.
(3) The methodology to develop complicated CMOS analog integrated circuits (IC) conceptual designs, specifically for undergraduate and graduate students.
(4) Development of complex conceptual designs using simple, non-expensive, and readily available software.
(5) Comparing a designed device with others published in the technical literature and used in biomedical instrumentation systems, particularly how far students are from major fundamental specifications and requirements such as gain, swing, frequency response, noise, and low power characteristics.
(6) Applying the designed amplifiers to CMOS-MEMS experimental devices in linear signal conditioning of multiplatform such as compound structures including Pirani, temperature, and gas sensors 42.

Finally, with the approach to teaching CMOS microelectronics and the use of readily available software such as Excel, it is possible to align teaching to reach the search for open innovation. 

## Figures and Tables

**Table 1:** Summary of CMOS N-Channel transistor large signal model.

	Long Channel L > 1 μm	Short Channel L < 1 μm
Ohmic	$i_{DS}=\frac{k’W}{L}\left[\left(V_{GS}-V_{T}\right)-\frac{V_{DS}}{2}\right]V_{DS}$	$i_{DS\;}=\frac{\mu_{e}C_{ox}E_{c}W}{\left(E_{c}L+V_{DS}\right)}\;[\;(V_{GS}-V_{T}$ $)\;V_{DS}-\frac{V_{DS}}{2}]V_{DS}$
$V_{DS}\left(sat\right)$	$V_{DS}\left(sat\right)=V_{GS}-V_{T}$	$V_{DS\left(sat\right)}=\frac{\left(V_{GS}-V_{T}\right)E_{c}L}{\left(V_{GS}-V_{T}\right)+E_{c}L}$
Saturation	$i_{DS}=\frac{k´W}{2L}{\left[V_{GS}-V_{T}\right]}^{2}$	$i_{D}=\frac{W\mu_{e}C_{ox}E_{c}}{2}\left[\frac{{\left(V_{GS}-V_{T}\right)}^{2}}{\left(V_{GS}-V_{T}\right)+E_{c}L}\right]$
Notes:	$k^{\prime }=\mu_{0}C_{ox}$	
	*E_c_* = critical horizontal field *E_y_* *μ_e_* = carrier mobility considering horizontal field *Ey*	

**Table 2:** MOSFET parameters for CMOS 0.5 μm process (C5_models) obtained in ON-SEMI Wafer Runs [6].

Parameter Symbol Description	Parameter Value			
		N-Channel	P-Channel	Units
*V_T_*_0_	Threshold voltage (*V_bs_* = 0)	0.76	−0.96	V
*K′*	Transconductance Parameter in saturation	115.6	37.8	$\mu A/V^{2}$
*γ*	Bulk threshold parameter	0.49	0.56	$V^{1/2}$
*λ*	Channel Modulation parameter	0.04	0.05	$V^{-1}$

**Table 3:** Resistance process parameters for CMOS 0.5 μm process used in ON-SEMI wafer runs [13].

Process Parameters	N+	P+	POLY	POLY2	POLY2 HR	N_well_	M1	M2	M3	UNITS
Sheet resistance	82.4	106.7	23.2	40.8	1076	808	0.09	0.09	0.05	Ω/sq
Contact resistance	59.6	152.5	16	26	-	-	0.84	0.84	0.82	Ω

**Table 4:** Capacitance process parameters for CMOS 0.5 μm process used in ON-SEMI wafer runs [15].

Capacitance Parameters	N+	P+	POLY	POLY2	M1	M2	M3	N_well_	UNITS
Area(substrate)	416	710	86	-	29	12	8	91	aF/μm^2^
Area(N+ active)	-	-	2456	-	-	-	-	-	aF/μm^2^
Area(P+ active)	-	-	2456	-	-	-	-	-	aF/μm^2^
Area(POLY)	-	-	-	922	64	16	9	-	aF/μm^2^
Area(POLY2)	-	-	-	-	58	-	-	-	aF/μm^2^
Area(M1)	-	-	-	-	-	32	12	-	aF/μm^2^
Area(M2)	-	-	-	-	-	-	32	-	aF/μm^2^
Fringe(substrate)	345	236	-	-	51	34	26	-	aF/μm^2^
Fringe(POLY)	-	-	-	-	70	39	28	-	aF/μm^2^
Fringe(M1)	-	-	-	-	-	49	33	-	aF/μm^2^
Fringe(M2)	-	-	-	-	-	-	55	-	aF/μm^2^
Overlap(N+active)	-	-	191	-	-	-	-	-	aF/μm^2^
Overlap(P+active)	-	-	234	-	-	-	-	-	aF/μm^2^

**Table 5:** Specifications and measured values from simulation tests in the CMOS Differential amplifier.

Specification	Requirement	Simulation Measured Value
CMOS process	0.5 μm	0.5 μm
Supply voltage	5 V rail to rail	±2.5 V
Supply current	>30 μA	49.2 μA
Gain	>30 dB	33.7 dB
Offset voltage	<20 mV	19.57 mV
f_-3dB_	$>100\;KH\;\left(C_{L}=2\;pF\right)$	812.8 KH
Slew Rate	$>10\;V/\mu s\;\left(C_{L}=2\;\;pF\right)$	−25 V/μs and+27 V/μs
Power Dissipation	<1 mW	0.492 mW

Note: Differences are between accepted tolerances of +/−10% for the conceptual model development.

**Table 6:** Specifications and measured values from simulation tests in the CMOS cascode amplifier using using 0.5 μ porocess.

Specification	Requirement	Simulation Measured Value
Supply voltage	5 V rail to rail	$V_{dd}=+5\;V\;and\;V_{ss}=0\;V$
Supply current	>30 μA	52 μA
Gain	** >26 dB **	31.1 dB
f_-3dB_	$>200\;KH\;\left(C_{L}=5\;pF\right)$	361 KH
Slew Rate	$>10\;V/\mu s\;\left(C_{L}=5\;pF\right)$	−300 V/μs and+10.45 V/μs
Output swing	from 1.5 to 4.0 V	from 0.66 to 4.03 V
Power Dissipation	<1 mW	0.996 mW

Note: Differences are between accepted tolerances of +/−10% for the conceptual model development.

**Table 7:** Specifications and measured values from simulation tests in the CMOS 2-stage OTA using 0.5 μ process.

Specification	Requirement	Simulation Measured Value
Supply voltage	5 V rail to rail	±2.5 V
Supply current	>30 μA	30 μA
Gain	>80 dB	80 dB
Gain-Bandwidth	>10 MH	14.7 MH
Slew Rate	$>10\;V/\mu s\;\left(C_{L}=2\;pF\right)$	−10 V/μs and+12 V/μs
ICMR	>±1.5 V	−2.3 to+2.4 V
Offset voltage	<±2 mV	−0.164 mV
PSRR	>70 dB	80 dB
Output Swing	>±2	−2.25 V and+2.25 V
Phase Margin	>70°	83°
Power Dissipation	<1 mW	0.896 mW

Note: Power dissipation includes the bias current for both stages.

**Table 8:** Specifications and measured values from simulation tests in the CMOS 3-stage op-amp using 0.5 μ process.

Specification	Requirement	Simulation Measured Value
Supply voltage	5 V rail to rail	±2.5 V
Supply current	>30 μA	73 μA
Gain	>90 dB	80.19 dB
Gain-Bandwidth	>10 MH	24.9 MH
Slew Rate	$>10\;V/\mu s\;\left(C_{L}=2\;pF\right)$	−9V/μs and+12.45 V/μs
ICMR	>±1.5 V	−2.2 to+1.84 V
Offset voltage	<±2 mV	0.036 mV
PSRR	>70 dB	80 dB
Output Swing	>±2	−2.25 V and+2.25 V
Phase Margin	>70°	126.4°
Power Dissipation	<1 mW	1.095 mW

Note: Power dissipation includes the bias current for the three stages.

**Table 9:** Proposed specifications for the op-amp design for biomedical applications.

Specification	Requirement	Comment
CMOS Process	0.5 μm	
Supply Voltage	From 2.0 V to 5.0 V rail to rail	+/−1.0 V to +/−2.5 V (for Vdd/Vss)
Supply Current	<100 μA	
Temperature range	0 to 70 °C	
Gain	>90 dB	
Gain Bandwidth	>10 MH	
Settling time	<0.5 μs	
Slew rate	>10 V/ μs	Average of SR+ and SR-
ICMR	>0.8 V to >+/−1.5 V	Depends upon the supply rails
PSRR	>70 dB	
Output swing	>+/−2 V	
Output resistance	<100 Ohms	
Offset Voltage	From +/−0.8 V to +/−2 mV	Depends upon the supply rails
Noise	<50 nV/sqrtH at 100 H	
PM	>70°	
Power dissipation	<1 mW	

**Table 10:** Transistor dimensions for the new three-stage conceptual design.

Transistor Dimensions (W/L) Considering L = 500 nm	
M1, M2	8
M3, M4	7
M5	5
M6	130
M7	22.5
M8	27
M9, M12	5
M10, M11	15

**Table 11:** Capacitors and resistors for the new three-stage conceptual design.

Cs are in pF and Rs are in KW	
$C_{L}$	2
$C_{C}$	0.5
$R_{Bias}$	265
$R_{z}$ (Implemented by M8)	5.22

**Table 12:** Comparison of specification, theoretical, schematic and layout results for the three-stage, low-power high-gain operational amplifier conceptual design.

Specification	Requirement	Theory	Schematic	Layout
		Calculation	Simulation	Simulation
$I_{SS}$	10 μA	10 μA	9.72 μA	9.72 μA
$A_{v0}$	>90 dB	134.3 dB	91.84 dB	91.72 dB
GB	>30 MH	40 M	50.87 M	46.1 M
$SR_{+}$	>20 V/μ	*	21.7 V/μ	21.1 V/μ
$SR_{-}$	>|20 V/μ|	*	19.6 V/μ	19.4 V/μ
PM	>70°	*	109.5°	93.9°
$P_{diss}$	<1 mW	0.403 mW	0.675 mW	0.661 mW
ICMR-	−0.8 V	−0.93 V	−0.816 V	−0.81 V
ICMR+	1.4 V	1.69 V	1.632 V	1.62 V
$V_{out}\left(max\right)$	1.4 V	1.61 V	1.35 V	1.34 V
$V_{out}\left(min\right)$	−1.4 V	−1.53 V	−1.35 V	−1.34 V
PSRR-	>70 dB	*	88 dB	88.1 dB
PSRR+	>70 dB	*	97 dB	97.9 dB
CMRR	>90 dB	*	128.8 dB	123.4 dB
Noise	$<50\;\mathrm{nV}/\sqrt{H}$	*	$20.9\;\mathrm{nV}/\sqrt{H}$	$20.9\;\mathrm{nV}/\sqrt{H}$

(*) no anticipated calculations were made for those parameters.

**Table 13:** Comparison of the three-stage op-amp conceptual design with Lópes-Martin´s design [35] which appears in the technical literature using CMOS 500 nm technology.

Parameter	Lópes-Martin et al. [35]	This Study
CMOS Technology μm	0.5	0.5
Supply V	±1.65	±1.8
Power consumption mW	0.26	0.67
Load capacitance pF	30	2
SR V/μs	2	20.19
$\mathrm{Silicon}\;\mathrm{area}\;{\mathrm{mm}}^{2}$	0.02	0.04
Gain bandwidth MH	1	46.06

**Table 14:** CMOS-MEMS signal conditioning for temperature sensor.

Temperature (°C)	V2 (V)	V1(V)	V2-V1 (V)	Gain (V/V)	Vref (V)	Vo (V)
−20	0.338	0.34	−0.002	100	0	−0.2
0	0.34	0.34	0	100	0	0
20	0.342	0.34	0.002	100	0	0.2
40	0.344	0.34	0.004	100	0	0.4
60	0.346	0.34	0.006	100	0	0.6

**Figure A1:**
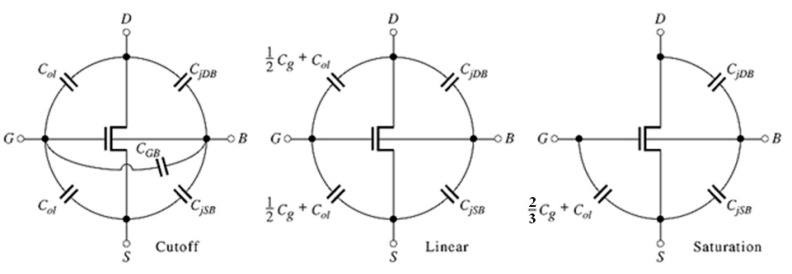
The parasitic capacitance model of the CMOS transistors.

## Appendix A

### Appendix A.1. Parasitic Capacitances Modeling

In the analysis of parasitics inside the CMOS transistor, we can identify three types of capacitances. First, the thin oxide capacitance between the gate and the other terminals of the transistor that depends on the operating zone with an approximate value of

(A1) $$C_{g}=WLC_{ox}$$

In the accumulation zone and factionary values of *Cg* of depletion, saturation, and linear zones of operation. The second type of parasitic capacitance inside the CMOS transistor is the PN junctions capacitors formed between the material P and N in the channel and substrate and sidewall capacitances. This is given by,

(A2) $$C_{j}≅\frac{C_{jb}\left(A_{b}+A_{sw}\right)}{\left(1-\frac{v_{j}}{\varphi_{B}}\right)}$$

The third type of parasitic capacitance inside a CMOS transistor is the Overlap capacitance that can be from the overlap of gates due to lateral diffusion (Cov) and fringing effects (Cf).

(A3) $$C_{ov}=C_{ox}\times L_{D}$$

(A4) $$C_{f}=\frac{2\varepsilon_{ox}}{\pi }\ln\left(1+\frac{T_{poly}}{t_{ox}}\right)$$

(A5) $$C_{ol}=C_{ov}+C_{f}$$

Table A1 illustrates the summary of the parasitic capacitances in a MOSFET at different operating zones. Moreover, Figure A1 depicts graphically the parasitic capacitance model of the CMOS devices.

**Table A1:** Parasitic MOSFET capacitance at different operation zones.

C/Zone	Cutoff	Linear	Saturation
C_gs_	C_ol_	C_ol_ + 0.5C_g_	C_ol_ + 0.67C_g_
C_gd_	C_ol_	C_ol_ + 0.5C_g_	C_ol_
C_gb_	[C_g_ C_jc_ ]/[C_g_ + C_jc_ ]	0	0
C_sb_	C_jsb_	C_jsb_	C_jsb_
C_db_	C_jdb_	C_jdb_	C_jdb_
Subscripts: sb = source bulk	gb = gate bulk	db = drain bulk	ol = overlapp

### Appendix A.2. Temperature Model and the Influence of Temperature in the Conceptual Design

In CMOS technology the influence of temperature in device conceptual designs is reflected mainly in the transconductance parameter and the threshold voltage, which can be evaluated using the Excel methods and validated using the “.temp” command in SPICE. The transconductance parameter changes with temperature as follows:

(A6) $$k\left(T\right)=k\left(T_{0}\right){\left[T/T_{0}\right]}^{-1.5}$$

The threshold voltage changes with temperature as follows:

(A7) $$V_{T}\left(T\right)≅V_{T}\left(T_{0}\right)+\propto \left(T-T_{0}\right)$$

Typically for NMOS transistors, $\propto_{NMOS}$ varies from −2 mV/°C to−3 mV/°C from 200 °K to 400 °K and for PMOS transistors the sign is reversed as one may expect. Therefore, the overall evaluations and calculations performed using the Excel method at room temperature would need to be re-calculated to account for the temperature range of operation. This can be validated using SPICE simulation as mentioned above.

For example, assume that we want to determine how the drain-source operating current of a CMOS 0.500 μm NMOS transistor varies from 27 °C to 100 °C. The device has W/L=5 μ/1 μ, $k\left(T_{0}\right)=117\;\mu A/V^{2}$, $V_{GS}=2\;V,\;V_{T}\left(T_{0}\right)=0.8$ and $T_{0}=27\;{}^{\circ}C$. The following calculations are performed:

(a) At room temperature:
  $$I_{DS}\left(27\;{}^{\circ}C\right)=\frac{117\mu A/V^{2}\;\cdot 5\mu }{2\cdot 1\mu }{\left(2-0.8\right)}^{2}=421.2\;\mu A$$
(b) At 100 °C (373 °K):
  $$k\left(100\;{}^{\circ}C\right)=117\mu A/V^{2}{\left(373/300\right)}^{-1.5}=84.39\;\mu A/V^{2}$$
  $$V_{T}\left(100\;{}^{\circ}C\right)=0.8-0.002\left(73{}^{\circ}C\right)=0.654\;V$$
  $$I_{DS}\left(100\;{}^{\circ}C\right)=\frac{84.39\mu A/V^{2}\;\cdot 5\mu }{2\cdot 1\mu }{\left(2-0.654\right)}^{2}=382.23\;\mu A$$

The previous example illustrates that a change of +73 °C in temperature produces a drop of 9.3% in the operating drain-source current of this device. Those calculations can be developed in the Excel methods to find the influence of temperature in the amplifier design. Those changes can also be verified by SPICE simulation using the temperature sweep accordingly.
